# Intermittent Hypoxia Training Prevents Deficient Learning-Memory Behavior in Mice Modeling Alzheimer's Disease: A Pilot Study

**DOI:** 10.3389/fnagi.2021.674688

**Published:** 2021-07-01

**Authors:** Myoung-Gwi Ryou, Xiaoan Chen, Ming Cai, Hong Wang, Marianna E. Jung, Daniel B. Metzger, Robert T. Mallet, Xiangrong Shi

**Affiliations:** ^1^Department of Medical Laboratory Science and Public Health, Tarleton State University, Texas A&M University System, Stephenville, TX, United States; ^2^Department of Pharmacology and Neuroscience, University of North Texas Health Science Center, Fort Worth, TX, United States; ^3^College of Sports Science, Jishou University, Jishou, China; ^4^College of Rehabilitation Sciences, Shanghai University of Medicine and Health Sciences, Shanghai, China; ^5^Department of Physiology and Anatomy, University of North Texas Health Science Center, Fort Worth, TX, United States

**Keywords:** Alzheimer's disease, beta-amyloid, BDNF, cerebral cortex, erythropoietin, intermittent hypoxia, memory-learning behavior

## Abstract

In mouse models of Alzheimer's disease (AD), normobaric intermittent hypoxia training (IHT) can preserve neurobehavioral function when applied before deficits develop, but IHT's effectiveness after onset of amyloid-β (Aβ) accumulation is unclear. This study tested the hypothesis that IHT improves learning-memory behavior, diminishes Aβ accumulation in cerebral cortex and hippocampus, and enhances cerebrocortical contents of the neuroprotective trophic factors erythropoietin and brain-derived neurotrophic factor (BDNF) in mice manifesting AD traits. Twelve-month-old female 3xTg-AD mice were assigned to untreated 3xTg-AD (*n* = 6), AD+IHT (*n* = 6), and AD+sham-IHT (*n* = 6) groups; 8 untreated wild-type (WT) mice also were studied. AD+IHT mice alternately breathed 10% O_2_ for 6 min and room air for 4 min, 10 cycles/day for 21 days; AD+sham-IHT mice breathed room air. Spatial learning-memory was assessed by Morris water maze. Cerebrocortical and hippocampal Aβ_40_ and Aβ_42_ contents were determined by ELISA, and cerebrocortical erythropoietin and BDNF were analyzed by immunoblotting and ELISA. The significance of time (12 vs. 12 months + 21 days) and treatment (IHT vs. sham-IHT) was evaluated by two-factor ANOVA. The change in swimming distance to find the water maze platform after 21 d IHT (−1.6 ± 1.8 m) differed from that after sham-IHT (+5.8 ± 2.6 m). Cerebrocortical and hippocampal Aβ_42_ contents were greater in 3xTg-AD than WT mice, but neither time nor treatment significantly affected Aβ_40_ or Aβ_42_ contents in the 3xTg-AD mice. Cerebrocortical erythropoietin and BDNF contents increased appreciably after IHT as compared to untreated 3xTg-AD and AD+sham-IHT mice. In conclusion, moderate, normobaric IHT prevented spatial learning-memory decline and restored cerebrocortical erythropoietin and BDNF contents despite ongoing Aβ accumulation in 3xTg-AD mice.

## Introduction

Normobaric intermittent hypoxia (IH) exposures can harm or protect the central nervous system, depending on the cumulative dose, frequency, and intensity of the hypoxia (Navarrete-Opazo and Mitchell, 2014). Brief cyclic bouts of high-intensity IH are applied up to 8–12 h/day in rodents to model sleep apnea and its sequelae (Tahawi et al., 2001; Phillips et al., 2004; Gozal et al., 2010). Mounting evidence (Kuo et al., 2020) identifies sleep apnea as a potential risk factor for Alzheimer's disease (AD). Indeed, imposition of 10-min cycles alternating intense normobaric hypoxia (5% O_2_) and 21% O_2_ for 8 h/day over 4 weeks increased cerebrocortical amyloid-β (Aβ) accumulation in 6-month-old transgenic mice with Alzheimer's disease (AD) traits (Shiota et al., 2013). In contrast to intense IH modeling sleep apnea, exposure of rats to 5–8 daily cycles alternating 5–10 min moderate, normobaric hypoxia (9.5–10% O_2_; ≤ 70 min hypoxia/session) and 4-min 21% O_2_ for ≤ 3 weeks protected brain from ethanol withdrawal excitotoxicity (Jung et al., 2008; Ryou et al., 2017). A 20-day IH training (IHT) regimen protected the brain from ethanol-withdrawal stress by dampening cerebrocortical presenilin-1 induction and Aβ accumulation (Ryou et al., 2017). However, the question remains whether normobaric IHT can preserve neurobehavioral function and attenuate Aβ accumulation in transgenic AD mice.

Amyloid β is produced by proteolytic processing of a transmembrane protein, amyloid precursor protein, by β- and γ-secretases (Ashall and Goate, 1994). Aβ_42_, admixed with smaller amounts of Aβ_40_, is the predominant Aβ species in extracellular amyloid plaques, which are implicated in AD pathogenesis (Sisodia and Price, 1995). Furthermore, Aβ_42_ is more fibrillogenic and neurotoxic than Aβ_40_ (Klein et al., 1998; Fernandez et al., 2014). Brain Aβ_42_ content is much higher in familial than sporadic AD, although neither Aβ_40_ content nor the ratio of Aβ_42_/Aβ_40_ differed between these AD subtypes (Dinkel et al., 2020).

Intermittent hypoxia exposures stimulate expression and synthesis of the growth/trophic factors erythropoietin (EPO) (Bernaudin et al., 2002) and brain-derived neurotrophic factor (BDNF) (Hassan et al., 2018). A powerful neuroprotectant (Rabie and Marti, 2008; Mallet and Ryou, 2017; Rey et al., 2019), EPO is expressed in astrocytes (Bernaudin et al., 2000; Ruscher et al., 2002) and neurons (Bernaudin et al., 2000, 2002) in response to hypoxemia. BDNF protects neurons (Yang et al., 2014) and promotes neuronal differentiation and growth, synapse formation and plasticity, cognitive functions and learning-memory (Yamada and Nabeshima, 2003; Zhu et al., 2010; Park and Poo, 2013). Although continuous hypobaric IH exposures can promote BDNF expression and improve cognitive performance (Zhu et al., 2010), the ability of cyclic normobaric IHT to augment the brain's EPO and BDNF expression in the setting of nascent AD, and whether IHT-enhanced EPO and BDNF expression is associated with improved learning-memory function, is unknown.

Currently, there are no treatments for AD in patients with established pathology. Recent reports in cognitively impaired elderly patients have shown IHT to be a potentially powerful intervention to preserve cognitive function (Wang et al., 2020), but IHT's salutary mechanisms, and its effectiveness against symptomatic AD, are unknown. This study tested the hypothesis that a 21-day normobaric IHT regimen improves learning-memory behavior, diminishes Aβ_40_ and Aβ_42_ accumulation in cerebral cortex and hippocampus, and enhances cerebrocortical EPO and BDNF contents in transgenic AD mice. Since progressive Aβ accumulation is an AD hallmark (Jagust, 2018), the IHT intervention was initiated when the mice were 12 rather than 6 (Shiota et al., 2013) or 9 months old (Romberg et al., 2011) to assess the impact of IHT after Aβ accumulation already had begun in hippocampus and cerebral cortex (Belfiore et al., 2019), challenging IHT's ability to mitigate ongoing AD pathology.

## Methods and Materials

### Animals

Female wild-type (WT) C57BL/B6 mice (*n* = 8) aged 11 months and female transgenic B6.Cg-PSEN1tm1Mpm with Tg-APPSwetauP301L AD mice (*n* = 18) aged 2 months were obtained from the Jackson Laboratory (JAX MMRRC Stock# 034830). The 3xTg-AD strain was selected because it is one of the most frequently studied transgenic AD models. Female 3xTg-AD mice were selected because it has been reported that “male transgenic mice may not exhibit the phenotypic traits” (descriptions: www.jax.org). By 12 months of age, Aβ plaques in these 3xTg-AD mice were significantly present in the brain (Belfiore et al., 2019). The mice were housed in the University of North Texas Health Science Center (UNTHSC) vivarium at 23 ± 1°C under a 24-h light (06:00–18:00) - dark (18:00–06:00) cycle. The animals were housed in groups of 4 or 5 in standard polycarbonate cages (28 × 17 × 12 cm) with corncob bedding and *ad libitum* access to food and water. AD mice were randomly assigned to one of three groups: 3xTg-AD pre-IHT control (*n* = 6), AD+IHT (*n* = 6; 21-day IHT program), or AD+sham-IHT (*n* = 6; 21-day sham IHT program). The study protocols were reviewed and approved by UNTHSC's Institutional Animal Care and Use Committee.

### Behavior Testing

Morris water maze (MWM) tests of spatial learning-memory behavior were conducted as described by Joshi et al. (2020). The MWM protocol was designed to compare the difference in the changes of the MWM performances between the AD+IHT and AD+sham-IHT mice after the interventions, which would provide the effect of IHT on learning-memory behavior in 3xTg-AD mice following the intervention. The tests were conducted between 9:00 and 12:00, after 2 pre-test trials were conducted to habituate the mice to the water temperature (24 ± 1°C) and the route to the northwest (NW) quadrant, where a 5 × 5 cm Plexiglas escape platform was placed and submerged 1.5 cm below the water surface. Mice were allowed to stay on the platform for ~10 s after reaching the platform during pre-test trials. The mouse was guided to the target site when the animal appeared to struggle to swim or when 3 min elapsed without finding the platform. The maze was divided into four equal quadrants, i.e., northeast (NE), southeast (SE), southwest (SW), and NW quadrants. To facilitate learning of the platform location, visual cues including geometric shapes and a scene from nature were posted on the laboratory walls around the maze (Brody and Holtzman, 2006). The water was made opaque with non-toxic white Colorations Powder Tempera Paint. The test trials started ~30 min after the animal completed pre-test trials. Animals started from the same point for pre-test trials and test trials, with the platform in the same location. All MWM test trials were continuously tracked using a computer-interfaced video system (Any-maze, Stoelting Co., Wood Dale, IL). Swimming duration, distance and speed were documented after releasing animals in the SE quadrant. The animals were dried with paper towels immediately after completing each test trial. Three test trials, separated by 5 min intervals, were conducted and the data averaged over the trials were reported. The test trials were completed within 20 min. All animals were able to independently locate the target site or platform after the pre-test trials.

Approximately 24 h after the MWM test, 8 WT and 6 AD mice (3xTg-AD control group) were weighed, anesthetized by ventilation with ~5% isoflurane, decapitated, and the brains harvested. The mice in the AD+IHT and AD+sham-IHT groups underwent 21-day IHT or sham-IHT interventions. A post-intervention MWM session was conducted 24 h after the last IHT or sham-IHT session. The mice were anesthetized and the brains harvested 24 h after the post-intervention MWM testing.

### Intermittent Hypoxia Training

**Figure 1:**
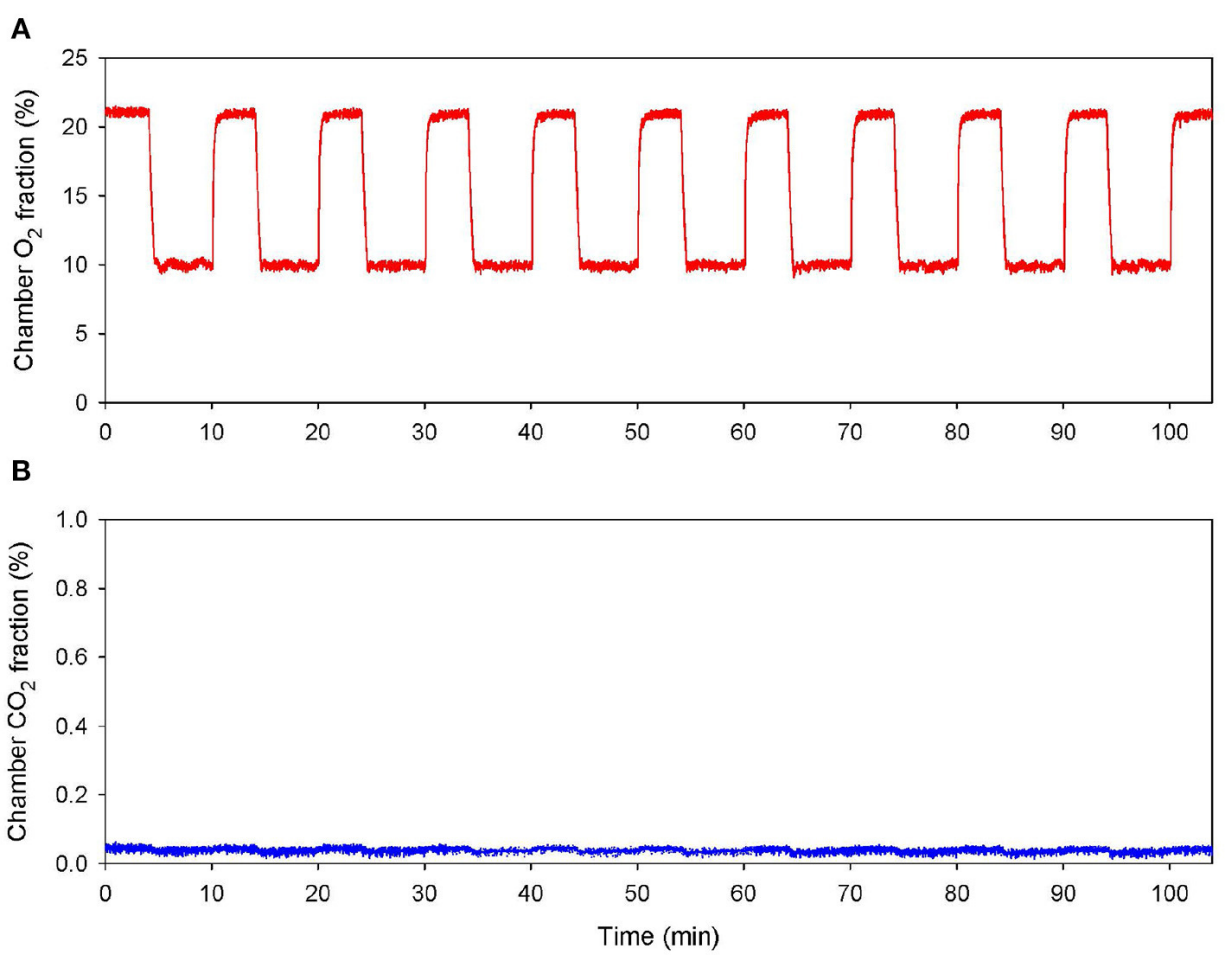
Normobaric intermittent hypoxia training session. Continuous record of the fractional concentrations of O_2_ **(A)** and CO_2_ **(B)** within the chamber during an IHT session.

Normobaric IHT was conducted after placing the home cages of the AD+IHT mice in a Plexiglas chamber (105 × 50 × 65 cm). Compressed 100% nitrogen gas was introduced into the chamber to lower O_2_ to 10% for 6 min, and then the chamber was opened to reintroduce room air for 4 min. Ten hypoxia-normoxia cycles were administered per daily session for 21 consecutive days. This IHT protocol combining moderate hypoxia intensity (10% O_2_) and duration (60 min cumulative hypoxia/session) was modified from a protocol that has proven to be cardio- (Zong et al., 2004; Mallet et al., 2006) and neuroprotective (Jung et al., 2008; Ryou et al., 2017) in animal models, and safe when applied to human subjects (Zhang et al., 2014; Wang et al., 2020). Fractions of O_2_ and CO_2_ in the chamber were continuously monitored throughout each IHT session (Figure 1) using a Perkin-Elmer (St. Louis, MO) model 1,100 mass spectrometer gas analyzer. The AD+sham-IHT mice were exposed to the room air adjacent to the IHT chamber. All IHT and sham-IHT sessions were carried out between 09:00 and 12:00.

### Brain Protein Extraction

Cerebral cortex and both hippocampi were excised, placed separately in labeled vials, flash-frozen in liquid N_2_, and stored at −80°C. Tissue proteins were extracted as described previously (Ju et al., 2012). Total protein concentrations in the extracts were measured by bicinchoninic acid assay (Thermo Fisher Scientific, Waltham, MA).

### Analyses of Cerebrocortical and Hippocampal Proteins

Total Aβ_40_ and Aβ_42_ contents in the cerebrocortical and hippocampal tissues were measured by enzyme-linked immunosorbent assay (ELISA) kits (Aβ_40_ Lot # KMB3481, Aβ_42_ Lot # KMB3441, Fisher Scientific, Hanover Park, IL) according to the manufacturer's instructions. Tissue extracts and Aβ_40_ and Aβ_42_ standards were incubated with mouse Aβ antibody at room temperature for 1 h. After thorough washing, anti-rabbit IgG horseradish peroxidase solution was added and incubated for 30 min at room temperature. After another washing, stabilized chromogen was added, followed by 30 min incubation at room temperature in the dark. Stop solution then was applied and absorbance was measured at 450 nm in a Spectramax Plus spectrophotometer (Molecular Devices, San Jose, CA).

Cerebrocortical contents of BDNF and EPO also were determined by ELISA (BDNF Lot # AB212166, EPO Lot # AB119593, Abcam, Cambridge, MA). BDNF (0–2,000 pg/ml) and EPO (0–3,000 pg/ml) standards were prepared by serial dilution. Extracts and standards were incubated in antibody-coated 96 well plates for 1 h at room temperature, washed with wash buffer, and then incubated with 3,3′,5,5′-tetramethylbenzidine substrate to develop color. BDNF and EPO protein contents were determined by spectrophotometry. BDNF, EPO, Aβ_40_, and Aβ_42_ concentrations were normalized against corresponding total protein concentrations.

Erythropoietin, BDNF and β-actin were analyzed by immunoblotting of cerebrocortical protein extracts as described previously (Ryou et al., 2012). Proteins (40 μg/lane) were separated by 10% SDS gel electrophoresis, and then electrophoretically transferred to polyvinylidene fluoride membranes (Millipore, Danvers, MA). Immunoblotting was performed with rabbit anti-BDNF (Abcam, Cambridge, MA; catalog #ab1083189, lot #GR3227037-4), goat anti-EPO (R&D Systems, Minneapolis, MN; catalog #SF959, lot #FIW0618078), and mouse anti-β-actin (Santa Cruz Biotechnology, Dallas, TX; catalog #sc-47778, lot #J2816) primary antibodies, followed by horseradish peroxidase-conjugated goat anti-rabbit (ImmunoReagents, Raleigh, NC; part #GtxRb-003-DHRPX, lot #20-170-011217), goat anti-mouse (ImmunoReagents; part #GtxMu-003-FHRPX, lot #58-98-021618) and mouse anti-goat IgG (Santa Cruz Biotechnology; catalog #sc-2354, lot #10871). Band densities were quantified with Doc-It Image Acquisition Software (UPV, Upland, CA) and normalized to β-actin density.

### Statistical Analysis

Single-factor analysis of variance (ANOVA) was applied to determine the group differences in body weight and MWM tests. Student's *t*-tests for two independent groups were applied to compare the baseline differences between the WT and 3xTg-AD groups and the post-intervention changes in the AD+IHT vs. AD+sham-IHT groups. Paired *t*-tests were applied to evaluate within-group changes in MWM performance post- vs. pre-intervention. Two-factor ANOVA was conducted to test the significance of time (12 months for untreated 3xTg-AD vs. 12 months plus 21 days for AD+IHT or AD+sham-IHT) and treatment (IHT vs. untreated 3xTg-AD and AD+sham-IHT) factors on the protein contents among the three 3xTg-AD groups. Duncan multiple range tests were applied *post-*hoc when ANOVA detected statistically significant effects of the major factor. Data are reported as group mean ± standard error of the mean. *P*-values < 0.05 were considered statistically significant, and *P*-values between 0.05 and 0.10 were taken to indicate trends. Statistical analyses were performed using Statistical Analysis System (SAS) software package version 9.4.

## Results

### Morris Water Maze Tests

**Figure 2:**
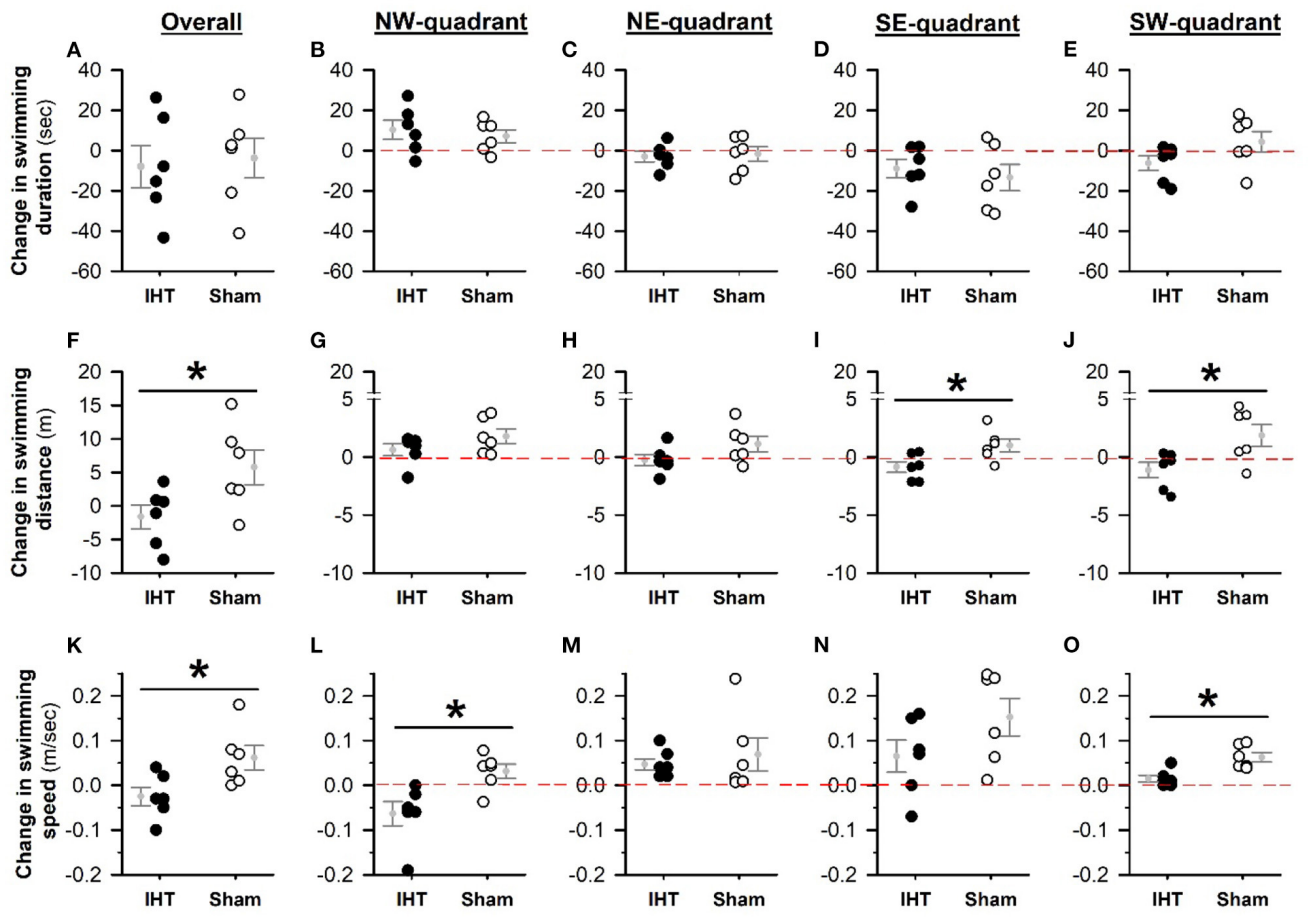
Changes in MWM tests from pre-treatment baseline. The changes in MWM performance variables (i.e., the differences between post- and pre-intervention values) are plotted for the IHT and sham-IHT interventions. A value below the zero line (the horizontal dashed line in each quadrant-specific graph) indicates a decrease in the variable after vs. before intervention, and a value above the zero line indicates an increase. Although changes in swimming duration after IHT vs. sham-IHT did not differ **(A)**, the change in swimming distance to find the submerged platform in the sham-IHT mice exceeded that of the IHT mice **(F)**. These between-group differences are significant in the SE **(I)** and SW **(J)** quadrants, but not in the target NW **(G)** and NE **(H)** quadrants. Swimming distance in the target (NW) quadrant increased in both the IHT and sham-IHT groups **(G)**. However, in the IHT group the changes in swimming distance in the other three quadrants are all below zero, but in the sham-IHT group are above zero **(H–J)**. The overall swimming speed **(K)** tended to increase after sham-IHT but not IHT, and this treatment effect was statistically significant in the target **(L)** and SW **(O)** quadrants. Individual data points and group mean ± standard error of the mean are plotted. *P < 0.05 for the indicated comparison.

Baseline MWM performance and body weight did not differ between the WT and 3xTg-AD mice (Table 1). The overall swimming duration to find the submerged platform placed in the NW quadrant did not decline significantly in the AD+IHT (−7.95 ± 10.49 s) or AD+Sham-IHT mice (−3.78 ± 9.82 s), and the changes in swimming duration did not differ between those two groups (Figure 2A). Furthermore, there were no significant group differences in the durations swum in any of the four quadrants (Figures 2B–E). However, the swimming distance to find the platform (Figure 2F) increased by 5.8 ± 2.6 m during the 21 d sham-IHT program (*P* = 0.077), and the change in swimming distance was greater (*P* < 0.05) than that of the IHT program (−1.6 ± 1.8 m). The two protocols also produced divergent effects (*P* < 0.05) on the distances swum in the opposite (SE) quadrant (AD+IHT: −0.9 ± 0.5 m vs. AD+sham-IHT: +1.0 ± 0.6 m) and the SW quadrant (AD+IHT: −1.12 ± 0.66 m vs. AD+sham-IHT: +1.87 ± 0.94 m).

**Table 1:** Results of Morris Water Maze test.

**Groups**		**WT**	**3xTg-AD**			**ANOVA *P-value***
			**Pre**	**IHT**	**Sham**	
Weight (g)		36.0 ± 1.9	39.2 ± 2.8	40.3 ± 3.4	43.0 ± 6.1	0.224
Overallswimming	Duration (s)	55.7 ± 9.2	48.6 ± 9.7	47.2 ± 10.3	60.6 ± 5.3	0.712
	Distance (m)	9.28 ± 1.69	9.39 ± 1.75	8.64 ± 1.75	6.63 ± 1.39	0.561
	Speed (m/s)	0.16 ± 0.01	0.19 ± 0.01	0.19 ± 0.01	0.14 ± 0.02	0.208
	Mobile-time (s)	50.6 ± 9.2	45.6 ± 9.8	47.0 ± 10.2	55.1 ± 4.9	0.886
Swimming in NW (target) quadrant	Duration (s)	14.8 ± 2.4	14.6 ± 3.1	10.6 ± 2.4	11.2 ± 2.6	0.363
	Distance (m)	2.24 ± 0.47	2.51 ± 0.49	2.08 ± 0.42	1.71 ± 0.34	0.766
	Speed (m/s)	0.15 ± 0.02	0.17 ± 0.02	0.19 ± 0.01	0.16 ± 0.02	0.149
Swimming in NE quadrant	Duration (s)	11.9 ± 2.4	10.6 ± 2.0	12.9 ± 4.6	24.2 ± 5.4	0.381
	Distance (m)	2.17 ± 0.42	2.16 ± 0.39	1.91 ± 0.42	1.63 ± 0.37	0.536
	Speed (m/s)	0.18 ± 0.01	0.21 ± 0.01	0.15 ± 0.04	0.10 ± 0.03	0.717
Swimming in SE quadrant	Duration (s)	17.2 ± 3.2	14.1 ± 2.7	14.5 ± 3.1	11.0 ± 3.5	0.252
	Distance (m)	2.88 ± 0.56	2.88 ± 0.54	2.91 ± 0.56	1.73 ± 0.42	0.530
	Speed (m/s)	0.16 ± 0.01	0.20 ± 0.01	0.20 ± 0.01	0.17 ± 0.42	0.370
Swimming in SW quadrant	Duration (s)	11.9 ± 2.0	9.4 ± 2.5	9.2 ± 2.4	14.2 ± 3.6	0.741
	Distance (m)	1.99 ± 0.35	1.84 ± 0.45	1.74 ± 0.38	1.57 ± 0.34	0.507
	Speed (m/s)	0.16 ± 0.01	0.20 ± 0.01	0.19 ± 0.01	0.15 ± 0.03	0.276
Mean distance from the target site (m)		0.47 ± 0.03	0.44 ± 0.03	0.49 ± 0.01	0.52 ± 0.04	0.687

*Single-factor analysis of variance (ANOVA) revealed no group differences in body weight and MWM performance between wild type (WT) mice and the 3xTg-AD groups. Values are group means ± standard error of the mean from 8 WT, 6 pre-treatment 3xTg-AD mice (designated “Pre”), 6 3xTg-AD mice completing IHT (“IHT”), and 6 3xTg-AD completing the sham-IHT program (“Sham”). NE, NW, SE, and SW denote northeast, northwest, southeast, and southwest MWM quadrants, respectively. The target platform was placed in the NW quadrant*.

Overall swimming speed (Figure 2K) was not altered by 21 d IHT (−0.03 ± 0.02 m/s) and tended to increase over 21 d sham-IHT (+0.06 ± 0.03 m/s, *P* = 0.08). The change of overall swimming speed in the IHT group differed from that of the sham-IHT group (*P* < 0.05). The changes in swimming speed in the target (NW) quadrant (Figure 2L) tended to decrease in the IHT group (−0.06 ± 0.03 m/s, *P* = 0.06), which was significantly slower (*P* < 0.05) than that in the sham-IHT group (0.03 ± 0.02 m/s), indicating that 21 d IHT intervention likely made the AD+IHT mice do more “thinking” than swimming during the MWM test, especially in the target quadrant. However, in the other three quadrants, the changes in swimming speed of the AD-IHT group were all above the zero line. In addition, the changes in swimming speed in the SW quadrant (Figure 2O) differed (*P* < 0.05) between the IHT group (0.02 ± 0.01 m/s) vs. the sham-IHT group (0.06 ± 0.01 m/s, *P* = 0.02). This increased swimming speed in the AD+sham-IHT group was associated with a greater swimming distance (Figure 2F) with no difference in swimming duration (Figure 2A) as compared to the AD+IHT group, suggesting spatial learning-memory decline, not physical impairment, occurred in the 3xTg-AD mice following 21 d sham-IHT.

Neither 21 d IHT nor sham-IHT produced significant changes in total mobile time during MWM tests (IHT: −13.3 ± 9.1 s vs. sham-IHT: +1.3 ± 7.4 s), and the respective changes did not differ (*P* = 0.24). Mean distance swimming around the MWM platform during the testing decreased (−0.15 ± 0.02 m, *P* = 0.01) following IHT, which was unchanged after sham-IHT (−0.06 ± 0.05 m, *P* = 0.23), suggesting that IHT intervention made the AD-IHT mice better oriented to and more concentrated on the target quarter during the MWM test. The difference between these responses was marginally significant (*P* = 0.10). Although body weight fell in the AD+IHT mice (−2.3 ± 0.8 g, *P* = 0.03) but not in the AD+sham-IHT group (−1.1 ± 1.0 g, *P* = 0.33), the weight changes over the course of IHT *vs*. sham-IHT did not differ (*P* = 0.39). None of the animals showed any physical impairment after 21-day IHT or sham-IHT.

### Aβ_40_ and Aβ_42_ Contents

**Figure 3:**
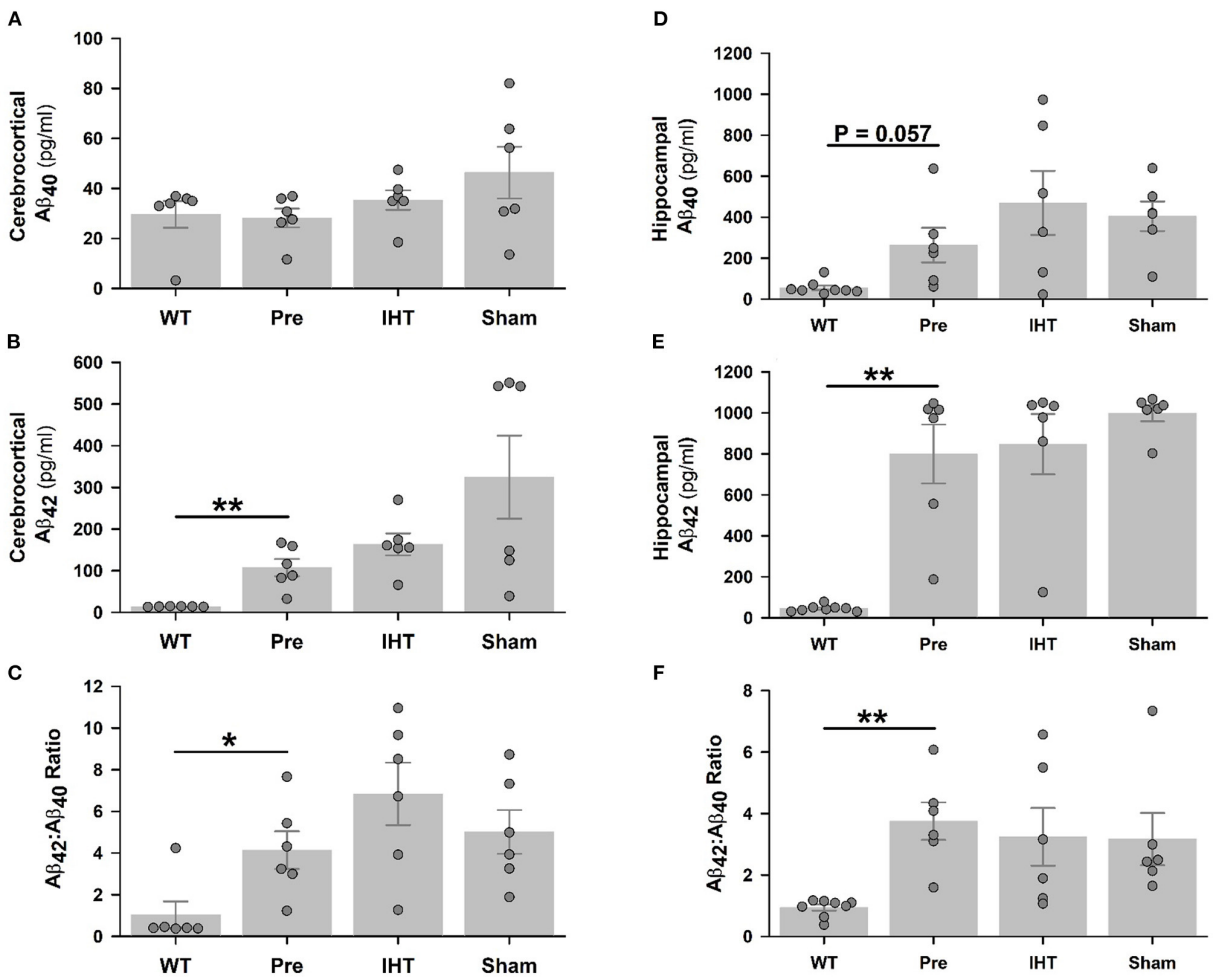
Total Aβ_40_ and Aβ_42_ contents determined by ELISA. Although cerebrocortical Aβ_40_ contents **(A)** were similar in the WT, 3xTg-AD, AD+IHT and AD+sham-IHT mice, cerebrocortical Aβ_42_ **(B)**, and the Aβ_42_/Aβ_40_ ratio **(C)** were greater in the 3xTg-AD than WT mice. Hippocampal Aβ_40_ content was marginally increased (*P* = 0.057) in the 3xTg-AD vs. WT mice **(D)**, and hippocampal Aβ_42_ **(E)** and the Aβ_42_/Aβ_40_ ratio **(F)** were greater in the 3xTg-AD than WT mice. Both the time factor (*P* = 0.087) and the treatment factor (*P* = 0.081) tended to significantly influence the cerebrocortical Aβ_42_ according to two-factor ANOVA. However, neither these factor altered the hippocampal Aβ_42_ vs. the pre-intervention 3xTg-AD group. Aβ_40_ contents or Aβ_42_/Aβ_40_ ratios in the cerebral cortex or hippocampus were not affected by the time or treatment factors. Individual data points and group mean ± standard error of the mean are plotted. *P < 0.05, **P < 0.01 for comparisons indicated by horizontal lines.

Baseline cerebrocortical Aβ_40_ contents (Figure 3A) were similar in the WT (28.1 ± 3.8 pg/ml) and 3xTg-AD mice (29.6 ± 5.3 pg/ml). Cerebrocortical Aβ_40_ contents in the 3xTg-AD groups were not affected by time (*P* = 0.127) or treatment factors (*P* = 0.165). However, cerebrocortical Aβ_42_ contents (Figure 3B) were nearly 8-fold greater (*P* < 0.01) in the 3xTg-AD (107.4 ± 20.7 pg/ml) than WT mice (13.7 ± 0.3 pg/ml). Cerebrocortical Aβ_42_ contents increased further (time factor *P* = 0.087) in the 22-day-older AD+IHT (163 ± 27 pg/ml) and AD+sham-IHT mice (325 ± 100 pg/ml) vs. the untreated 3xTg-AD mice, and IHT tended to dampen Aβ_42_ accumulation (treatment factor *P* = 0.081) vs. sham-IHT.

Hippocampal Aβ_40_ content (Figure 3D) was 5-fold greater (*P* = 0.057) in the 3xTg-AD (263 ± 85 pg/ml) vs. WT mice (55 ± 12 pg/ml), while Aβ_42_ content (Figure 3E) was almost 18-fold greater (*P* < 0.01) in the 3xTg-AD (799 ± 144 pg/ml) than WT mice (45 ± 5 pg/ml). Neither hippocampal Aβ_40_ nor Aβ_42_ contents in the three 3xTg-AD groups were affected by the time or treatment factors. Cerebrocortical (Figure 3C) and hippocampal (Figure 3F) Aβ_42_:Aβ_40_ content ratios mirrored Aβ_42_ contents.

### Erythropoietin and BDNF Contents

**Figure 4:**
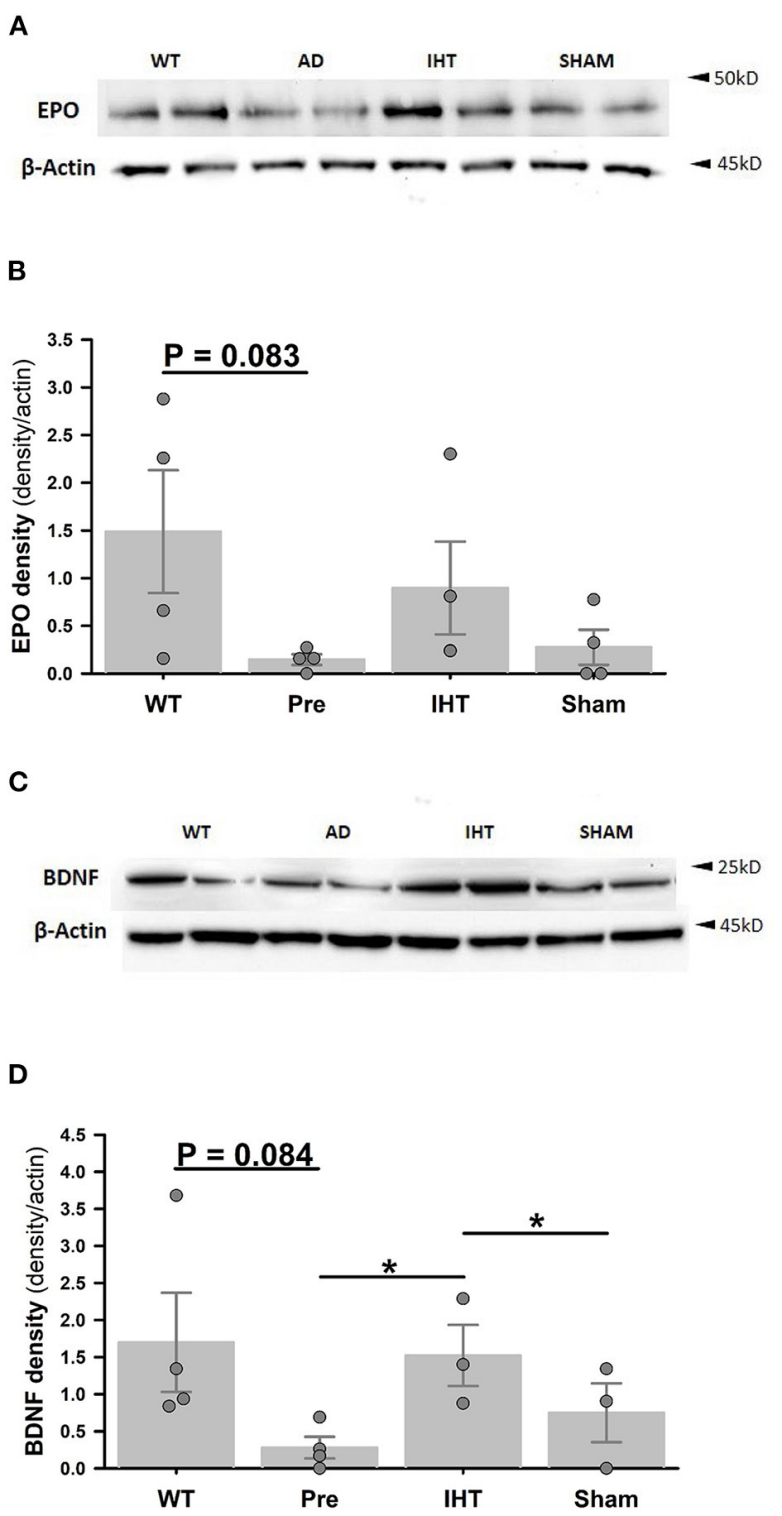
Cerebrocortical erythropoietin and BDNF immunoblots. **(A)** Cerebrocortical erythropoietin (EPO) and β-actin immunoblots. **(B)** EPO band densities normalized to β-actin band densities. **(C)** Cerebrocortical BDNF and β-actin immunoblots. **(D)** BDNF densities normalized to β-actin band densities. Analyses of the band densities indicated that cerebrocortical EPO and BDNF contents were lower in the 3xTg-AD vs. WT mice. IHT, but not sham-IHT, tended to restore EPO and BDNF contents in the 3xTg-AD mice. For EPO and BDNF contents, treatment factors are *P* = 0.257 and *P* = 0.003, respectively, and time factors are *P* = 0.217 and *P* = 0.004, respectively. *P < 0.05 for comparisons indicated by horizontal lines. Individual data points and group mean ± standard error of the mean are shown.

Immunoblotting (Figure 4) revealed sharply lower β-actin-normalized cerebrocortical EPO (Figure 4B: *P* = 0.083) and BDNF (Figure 4D: *P* = 0.084) contents in the untreated 3xTg-AD mice (EPO: 0.15 ± 0.06; BDNF: 0.28 ± 0.15) vs. WT mice (EPO: 1.49 ± 0.64; BDNF: 1.70 ± 0.67). In the post-treatment groups, β-actin-normalized EPO contents (Figure 4B: AD+IHT: 0.90 ±0.49; AD+sham-IHT: 0.27 ± 0.18) were not significantly affected by time factor (*P* = 0.217) or treatment factor (*P* = 0.257). However, BDNF contents (Figure 4D) were significantly influenced by both time (*P* = 0.004) and treatment factors (*P* = 0.003). β-actin-normalized BDNF contents (Figure 4D) in the AD+IHT mice (1.52 ± 0.14) were doubled vs. AD+sham-IHT mice (0.75 ± 0.62) and increased over 5-fold vs. pre-treated 3xTg-AD (0.28 ± 0.15).

**Figure 5:**
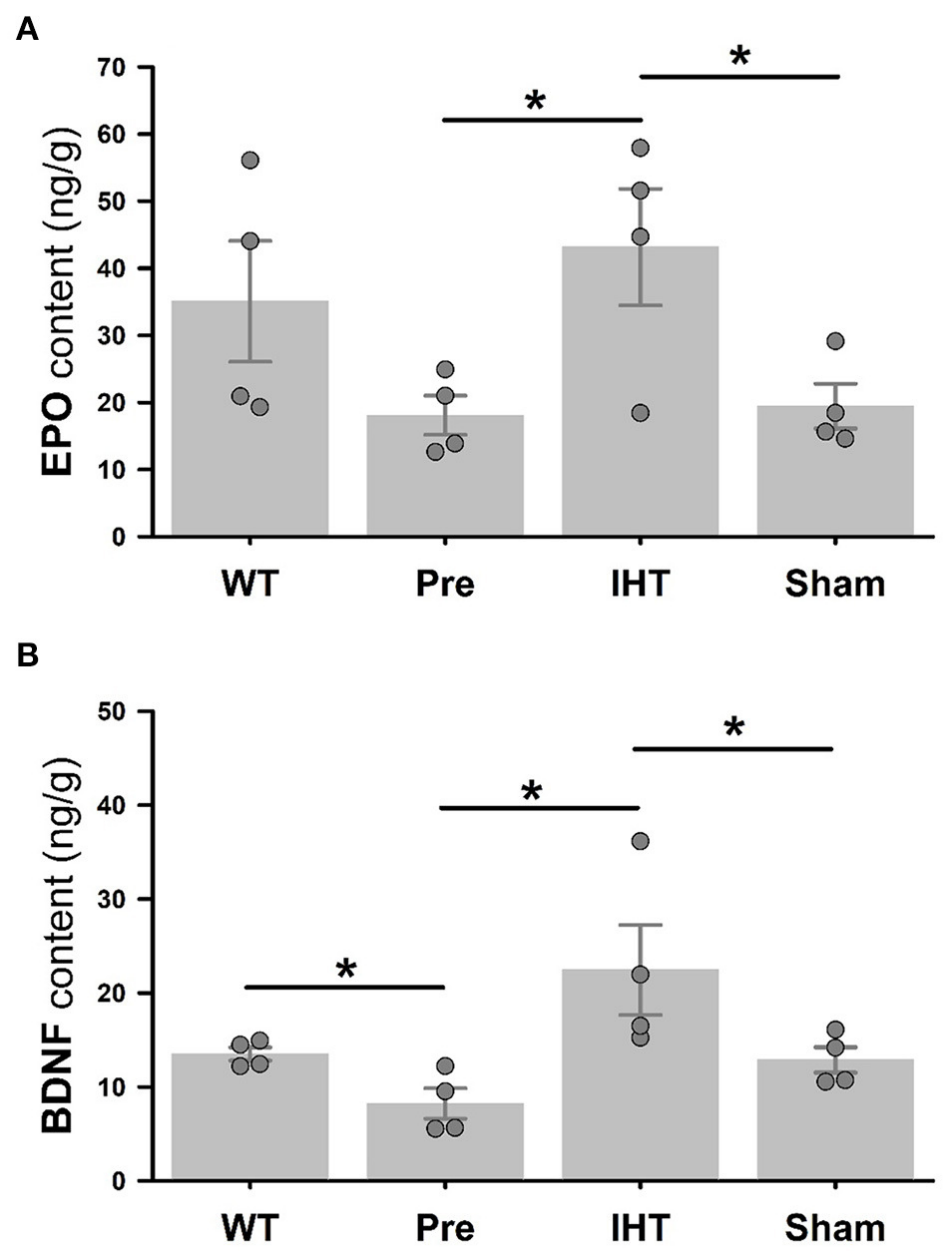
ELISA analyses of cerebrocortical EPO and BDNF. Cerebrocortical EPO **(A)** and BDNF **(B)** contents are significantly greater in the AD+IHT than pre-intervention 3xTg-AD mice. For EPO and BDNF contents, treatment factors are *P* = 0.008 and *P* = 0.001, respectively, and time factors are *P* = 0.047 and *P* = 0.002, respectively. *P < 0.05 for comparisons indicated by horizontal lines. Individual data points and group mean ± standard error of the mean are shown.

Figure 5 presents cerebrocortical EPO (panel A) and BDNF (panel B) contents (ng/g tissue) measured by ELISA. Although the difference in EPO contents (Figure 5A) in WT (35.1 ± 9.0) vs. 3xTg-AD mice (18.1 ± 2.9) did not attain statistical significance (*P* = 0.123), BDNF content (Figure 5B) was greater (*P* < 0.05) in the WT (13.5 ± 0.7) vs. 3xTg-AD mice (8.2 ± 1.6). Both EPO and BDNF contents were affected by the time (EPO: *P* = 0.047; BDNF: *P* = 0.002) and treatment factors (EPO: *P* = 0.008; BDNF: *P* = 0.001). *Post-hoc* analysis confirmed that 21-day IHT increased cerebrocortical EPO and BDNF contents. Thus, EPO and BDNF contents (ng/g tissue) in the AD+IHT group (EPO: 43.1 ± 8.7; BDNF: 22.4 ± 4.8) exceeded those in the pre-treatment 3xTg-AD mice (EPO: 18.1 ± 2.9; BDNF: 8.2 ± 1.6). EPO content (Figure 5A) in the AD+IHT mice also exceeded that of the AD+sham-IHT mice (19.3 ± 3.3), and a trend toward higher BDNF content in the AD+IHT vs. AD+sham-IHT mice (12.9 ± 1.4) was noted (Figure 5B).

## Discussion

This study demonstrated that a 21-day normobaric IHT intervention, initiated after the onset of cerebrocortical and hippocampal Aβ_42_ accumulation, prevented declination of spatial learning-memory function in transgenic mice predisposed to develop AD-like neurobehavioral impairments. Specifically, in the MWM test the overall swimming distance to find a submerged platform was significantly shorter in the AD+IHT than the AD+sham-IHT mice, and the IHT intervention made the AD+IHT mice better oriented to the target quarter. Notably, IHT effected this improvement without significantly dampening Aβ_40_ accumulation in the cerebral cortex and hippocampus, although the cerebrocortical Aβ_42_ content tended to be diminished (treatment factor *P* = 0.081) in the AD+IHT as compared to the AD+sham-IHT mice following 21-day interventions. Instead, the functional improvement may be ascribable, at least in part, to IHT-induction of the neuroprotective trophic/growth factors EPO and BDNF.

### Learning-Memory vs. Amyloid-β in 3xTg-AD Mice

Previously, we reported that a 20-day normobaric IHT program protected the brain from ethanol-withdrawal stress by dampening cerebrocortical presenilin-1 overexpression and Aβ_40_ and Aβ_42_ accumulation in 4 month-old, non-AD rats (Ryou et al., 2017). Here, however, cerebrocortical and hippocampal Aβ contents were not affected significantly by the IHT intervention. Although the two studies were conducted in different species, the fact that the mice already were 12 months old at the start of the IHT program may account for the lack of an IHT effect on Aβ content. The older brain may have a reduced plasticity or adaptability, dampening its responsiveness to IHT. Also, unlike the rats, the mice were predisposed genetically to develop hallmarks of AD. The accumulated Aβ_42_ in the 3xTg-AD mice may be innately more difficult to eradicate than the Aβ accumulation in rats experiencing ethanol withdrawal (Ryou et al., 2017).

Despite similar cerebrocortical and hippocampal Aβ contents (Figure 3), the spatial learning-memory behavior of AD mice appeared to be improved by IHT vs. sham-IHT (Figure 2). This finding is concordant with a recent longitudinal study showing no correlation of amyloidosis with cognitive outcome in transgenic AD mice (Focke et al., 2019). Arguably, Aβ content might not be a quantitatively sensitive predictor of the changes in learning-memory function in transgenic AD mice (Foley et al., 2015). Although cerebrocortical Aβ_42_ contents tended to be lower (*P* = 0.081) in the AD+IHT than AD+sham-IHT mice, there was no intervention effect on hippocampal Aβ_42_ contents between these two groups. Therefore, IHT induction of the neuroprotective trophic/growth factors EPO and BDNF could constitute a mechanism improving learning-memory function in the AD+IHT mice.

### Trophic Factors and Neuroprotection

The specific mechanism of IHT-induced brain protection or recovery is not fully understood, but the hypoxia-responsive transcription factor, hypoxia-inducible factor (HIF) appears to play a key role in the transition from the acute to second phases of cellular adaptation to various neuropathological conditions (Semenza, 2009; Lukyanova et al., 2013; Dengler et al., 2014; Rybnikova and Samoilov, 2015). In the acute phase, cytoprotection is effected by regulating ion channel permeability, protein phosphorylation, and post-translational modification (Li et al., 2017). HIF activation, specifically stabilization of the O_2_-regulated HIF-1α subunit, promotes expression of key elements of the second phase of adaptation, including EPO, BDNF, antioxidant enzymes, and anti-apoptotic proteins (Rybnikova and Samoilov, 2015; Vetrovoy et al., 2017).

Erythropoietin has been shown to protect neurons from ischemic damage (Sakanaka et al., 1998; Malhotra et al., 2006; Ratilal et al., 2014), suppress neuronal apoptosis and necroptosis (Rabie and Marti, 2008; Mallet and Ryou, 2017) by attenuating cytokine production and inflammation (Villa et al., 2003), reduce astrocyte edema in stroke (Gunnarson et al., 2009), and promote brain angiogenesis after focal ischemia (Li et al., 2007). EPO-loaded lipid nanoparticles restored memory and cognitive functions of AD mice (Dara et al., 2019). The present study demonstrated that moderate, normobaric IHT increased endogenous EPO content in the cerebral cortex of 3xTg-AD mice. The increased EPO content after 21-day normobaric IHT was associated with improved MWM performance in the IHT-treated vs. sham-IHT AD mice.

Brain content of the neurotrophin BDNF is decreased in AD, Huntington disease and Parkinson's disease (Hock et al., 2000; Zuccato et al., 2008; Miranda et al., 2019). In AD, the greatest declines in BDNF are in the hippocampus and the parietal, entorhinal, and frontal cortices (Hock et al., 2000). In the present study, cerebrocortical BDNF content was indeed lower in the 3xTg-AD than wild-type mice. Nonetheless, this AD-related difference could be reversed by 21-day normobaric IHT, which markedly increased cerebrocortical BDNF and EPO contents vs. those of untreated and sham-IHT-treated 3xTg-AD mice. Collectively, augmented expression of these neuroprotective trophic/growth factors could contribute to improved learning-memory behavior in the 3xTg-AD mice.

### Relationship Between EPO and BDNF Expression

The molecular signaling mechanisms mediating the parallel increases in cerebrocortical erythropoietin and BDNF contents in the IHT-treated 3xTg-AD mice are not yet known. In particular, evidence is equivocal regarding the hierarchy between EPO and BDNF expression. Exogenous BDNF increased EPO expression and signaling in rat cortical neurons (Wu et al., 2010), and pharmacological induction of BDNF expression activated EPO formation and suppressed inflammation in a murine microglial cell line (Lai et al., 2018). On the other hand, EPO increased BDNF content in rat brain following embolic stroke (Wang et al., 2004) and in neurotoxin-challenged rat hippocampal neurons (Viviani et al., 2005), increased BDNF content and anti-apoptotic signaling in brains of haloperidol-challenged rats (Pillai et al., 2008), and augmented hippocampal BDNF content and preserved spatial and fear memory in aged rats (Jia et al., 2016). EPO mobilization of intracellular Ca^2+^ sequentially activates calcium-calmodulin dependent protein kinase and CREB-1, the principal promoter of BDNF gene transcription (Viviani et al., 2005; Song et al., 2015), yet BDNF-activated kinases also mobilize CREB-1 in mouse cortical neurons, potentially intensifying BDNF formation (Kim et al., 2017). Further research is essential to decipher the potentially reciprocal inductions of EPO and BDNF, and their contributions to IHT-induced cognitive improvements in animal models of AD.

### Importance of IH Dose

Hypoxia intensity or inspired fraction of O_2_ (F_I_O_2_) and cumulative exposure duration are the major factors that determine if IH exposures are potentially detrimental or beneficial. Previously, Shiota et al. reported a significant increase in Aβ_42_ content (with no changes in Aβ_40_ content and MWM performance) in 6-month-old male 3xTg-AD mice following the intervention of 10-min exposure to intense (5% O_2_) normobaric hypoxia interspersed with 10-min 21% O_2_ for 8 h/day over 4 weeks (Shiota et al., 2013). Recently, Arias-Cavieres et al. (2020) reported diminished synaptic long-term potentiation (LTP) in mouse hippocampus and impaired Barnes maze performance after IH exposures of 1-min ~4.5% O_2_ and 5-min 21% O_2_ for 8 h/day over 10 days. This adverse outcome was ascribed to over-accumulation of reactive oxygen species (ROS) mediated by excessive HIF-1α mobilization. In contrast, Jung et al. found 5–8 daily cycles of 5–10 min hypoxia (F_I_O_2_ 9.5–10%) and 4-min normoxia over 20 days mitigated oxidative stress and behavior deficits in rats during abrupt ethanol-withdrawal following 35-day ethanol intoxication (Jung et al., 2008). Ryou et al. reported a similar IH protocol in ethanol-withdrawn rats prevented cerebrocortical Aβ_40_ and Aβ_42_ accumulation, probably by moderating ethanol withdrawal-induced γ-secretase overactivation and oxidative damage (Ryou et al., 2017). In the IH studies of Jung et al. (2008) and Ryou et al. (2017), cumulative hypoxia (F_I_O_2_ 9.5–10%) durations were 25–70 min per daily session and 483 min or ~8 h over the 20-day IHT program, well below the dose and intensity of IH exposures modeling sleep apnea (Shiota et al., 2013; Arias-Cavieres et al., 2020).

In dogs, moderate IH exposures eliciting cardioprotective adaptations lowered the partial pressure of arterial O_2_ (PaO_2_) to 45–50 mmHg (Mallet et al., 2006). In humans, similar PaO_2_ values lower arterial O_2_ saturation from 97 to 70–75% (Zhang et al., 2014) and cerebral tissue O_2_ saturation from 70–75 to 50–55% (Liu et al., 2017). During such IH exposures, moderate hypoxemia-induced cerebral vasodilation maintains adequate O_2_ delivery to meet the brain's metabolic demands (Liu et al., 2017). However, intense IH exposures to simulate sleep apnea may induce more severe hypoxemia, e.g., PaO_2_ *c*. 35 mmHg (Wall et al., 2014). Although such intense IH elicits robust HIF-1α activation and EPO expression (Wall et al., 2014), it likely imposes tissue hypoxia so severe that O_2_ supply falls below neuronal metabolic demand, injuring the central nervous system.

### Study Limitations and Perspectives

Because the hippocampus is a center of learning and memory, the lack of EPO and BDNF data in that region precludes definitive conclusions regarding the mechanisms of IHT-improved learning-memory behavior in the 3xTg-AD mice. Hyperphosphorylated tau, which like Aβ accumulates in brains of AD patients, was not measured in this study. Tau hyperphosphorylation, although possibly not fully manifest at 12 months in the 3xTg-AD mice (Belfiore et al., 2019), could be strongly associated with learning-memory impairment. However, it should be noted that amyloid β accumulation precedes tau hyperphosphorylation in 3xTg-AD mice (Belfiore et al., 2019); indeed, amyloid β creates the inflammatory milieu that activates the tau-phosphorylating protein kinases (Ontiveros-Torres et al., 2016; Huber et al., 2018). Since IHT did not significantly lower amyloid β content in the cerebral cortex or hippocampus, it could be postulated that IHT may not have altered tau phosphorylation in the present study. Nonetheless, evaluation of tau phosphorylation and hippocampal EPO and BDNF contents in IHT- vs. sham-IHT-treated 3xTg-AD mice, and analysis of IHT's impact on neuronal injury and death in the 3xTg-AD mice, are appropriate extensions of this work. Furthermore, additional cognitive behavioral assessments such as the Barnes maze test, the Y-maze test, and the Novel object-recognition test should be applied to support the favorable effects of IHT on the MWM assessment of spatial learning behavior. Neuroimaging studies of cerebral cortex and hippocampus could determine if IHT-augmentation of EPO and BDNF is associated with structural adaptations, e.g., *de novo* neurogenesis (Shingo et al., 2001; Zhu et al., 2010).

Although the persistence of the IHT-induced benefits in 3xTg-AD mice is not yet known, IHT-related improvements in learning-memory behavior and augmented expression of neuroprotective growth/trophic factors may persist well beyond completion of the IHT intervention. In a rat model of ethanol intoxication-withdrawal, an IHT regimen similar to the present one sharply attenuated neurobehavioral deficits during acute ethanol withdrawal, and this protection remained effective against a second ethanol withdrawal 5 weeks after completing IHT (Ju et al., 2012). Long-term assessments of spatial learning-memory and brain Aβ, EPO and BDNF contents beyond 24 h post-IHT are appropriate goals of future studies.

## Conclusions

A 3-week moderate, normobaric IHT regimen stabilized neurobehavioral function and induced expression of the neuroprotective trophic factors EPO and BDNF, without lowering already-accumulated amyloid-β, in transgenic mice modeling AD. The IHT regimen augmented cerebrocortical EPO and BDNF contents, thereby reversing the depletion of these neuroprotective trophic/growth factors in 3xTg-AD mice, in the face of ongoing Aβ accumulation. Thus, IHT can activate cerebrocortical EPO and BDNF formation even after the onset of AD pathogenesis. The enhanced EPO and BDNF contents were associated with improved spatial learning-memory function in the 3xTg-AD mice vs. age-matched controls without IHT intervention. This study in transgenic mice displaying the AD phenotype provides the first empirical evidence that IHT initiated after AD onset can prevent cognitive decline, identifies potential molecular underpinnings of this cognitive improvement, and supports the possibility that moderate, normobaric IHT regimens could be effectively applied for treating AD after the onset of Aβ_42_ accumulation.

## Data Availability Statement

The raw data supporting the conclusions of this article will be made available by the corresponding author upon request.

## Ethics Statement

The animal study was reviewed and approved by Institutional Animal Care and Use Committee at UNTHSC.

## Author Contributions

M-GR, XC, MC, HW, MJ, RM, and XS: conceived and planned experiments. M-GR, XC, and XS: analyzed data. M-GR, MJ, RM, and XS: interpreted results of experiments, prepared figures and drafted manuscript, and edited the manuscript. All authors: performed experiments and approved final version of manuscript.

## Conflict of Interest

The authors declare that the research was conducted in the absence of any commercial or financial relationships that could be construed as a potential conflict of interest.

## References

[B1] Arias-Cavieres A. Khuu M. A. Nwakudu C. U. Barnard J. E. Dalgin G. Garcia A. J. III. (2020). A HIF1a-dependent pro-oxidant state disrupts synaptic plasticity and impairs spatial memory in response to intermittent hypoxia. eNeuro 7:ENEURO.0024-20.2020. 10.1523/ENEURO.0024-20.2020 32493757 PMC7363479

[B2] Ashall F. Goate A. M. (1994). Role of the beta-amyloid precursor protein in Alzheimer's disease. Trends Biochem. Sci. 19, 42–46. 10.1016/0968-0004(94)90173-2 8140621

[B3] Belfiore R. Rodin A. Ferreira E. Velazquez R. Branca C. Caccamo A.. (2019). Temporal and regional progression of Alzheimer's disease-like pathology in 3xTg-AD mice. Aging Cell 18:e12873. 10.1111/acel.12873 30488653 PMC6351836

[B4] Bernaudin M. Bellail A. Marti H. H. Yvon A. Vivien D. Duchatelle I.. (2000). Neurons and astrocytes express EPO mRNA: oxygen-sensing mechanisms that involve the redox-state of the brain. Glia 30, 271–278. 10.1002/(SICI)1098-1136(200005)30:3<271::AID-GLIA6>3.0.CO;2-H 10756076

[B5] Bernaudin M. Nedelec A. S. Divoux D. MacKenzie E. T. Petit E. Schumann-Bard P. (2002). Normobaric hypoxia induces tolerance to focal permanent cerebral ischemia in association with an increased expression of hypoxia-inducible factor-1 and its target genes, erythropoietin and VEGF in the adult mouse brain. J. Cereb. Blood Flow Metab. 22, 393–403. 10.1097/00004647-200204000-00003 11919510

[B6] Brody D. L. Holtzman D. M. (2006). Morris water maze search strategy analysis in PDAPP mice before and after experimental traumatic brain injury. Exp. Neurol. 197, 330–340. 10.1016/j.expneurol.2005.10.020 16309676 PMC1913184

[B7] Dara T. Vatanara A. Sharifzadeh M. Khani S. Vakilinezhad M. A. Vakhshiteh F.. (2019). Improvement of memory deficits in the rat model of Alzheimer's disease by erythropoietin-loaded solid lipid nanoparticles. Neurobiol. Learn. Mem. 166:107082. 10.1016/j.nlm.2019.107082 31493483

[B8] Dengler V. L. Galbraith M. Espinosa J. M. (2014). Transcriptional regulation by hypoxia inducible factors. Crit. Rev. Biochem. Mol. Biol. 49, 1–15. 10.3109/10409238.2013.838205 24099156 PMC4342852

[B9] Dinkel F. Trujillo-Rodriguez D. Villegas A. Streffer J. Mercken M. Lopera F.. (2020). Decreased deposition of beta-amyloid 1-38 and increased deposition of beta-amyloid 1-42 in brain tissue of presenilin-1 e280a familial Alzheimer's disease patients. Front. Aging Neurosci. 12:220. 10.3389/fnagi.2020.00220 32848702 PMC7399638

[B10] Fernandez M. A. Klutkowski J. A. Freret T. Wolfe M. S. (2014). Alzheimer presenilin-1 mutations dramatically reduce trimming of long amyloid β-peptides (Aβ) by γ-secretase to increase 42-to-40-residue Aβ. J. Biol. Chem. 289, 31043–31052. 10.1074/jbc.M114.581165 25239621 PMC4223309

[B11] Focke C. Blume T. Zott B. Shi Y. Deussing M. Peters F.. (2019). Early and longitudinal microglial activation but not amyloid accumulation predicts cognitive outcome in PS2APP mice. J. Nucl. Med. 60, 548–554. 10.2967/jnumed.118.217703 30262517

[B12] Foley A. M. Ammar Z. M. Lee R. H. Mitchell C. S. (2015). Systematic review of the relationship between amyloid-β levels and measures of transgenic mouse cognitive deficit in Alzheimer's disease. J. Alzheimers Dis. 44, 787–795. 10.3233/JAD-142208 25362040 PMC4346318

[B13] Gozal D. Nair D. Goldbart A. D. (2010). Physical activity attenuates intermittent hypoxia-induced spatial learning deficits and oxidative stress. Am. J. Respir. Crit. Care Med. 182, 104–112. 10.1164/rccm.201001-0108OC 20224062 PMC2902754

[B14] Gunnarson E. Song Y. Kowalewski J. M. Brismar H. Brines M. Cerami A.. (2009). Erythropoietin modulation of astrocyte water permeability as a component of neuroprotection. Proc. Natl. Acad. Sci. U. S. A. 106, 1602–1607. 10.1073/pnas.0812708106 19164545 PMC2629445

[B15] Hassan A. Arnold B. M. Caine S. Toosi B. M. Verge V. M. K. Muir G. D. (2018). Acute intermittent hypoxia and rehabilitative training following cervical spinal injury alters neuronal hypoxia- and plasticity-associated protein expression. PLoS ONE 13:e0197486. 10.1371/journal.pone.0197486 29775479 PMC5959066

[B16] Hock C. Heese K. Hulette C. Rosenberg C. Otten U. (2000). Region-specific neurotrophin imbalances in Alzheimer disease: decreased levels of brain-derived neurotrophic factor and increased levels of nerve growth factor in hippocampus and cortical areas. Arch. Neurol. 57, 846–851. 10.1001/archneur.57.6.846 10867782

[B17] Huber C. M. Yee C. May T. Dhanala A. Mitchell C. S. (2018). Cognitive decline in preclinical Alzheimer's disease: amyloid-beta versus tauopathy. J. Alzheimers Dis. 61, 265–281. 10.3233/JAD-170490 29154274 PMC5734131

[B18] Jagust W. (2018). Imaging the evolution and pathophysiology of Alzheimer disease. Nat. Rev. Neurosci. 19, 687–700. 10.1038/s41583-018-0067-3 30266970 PMC7032048

[B19] Jia Z. Xue R. Ma S. Xu J. Guo S. Li S.. (2016). Erythropoietin attenuates the memory deficits in aging rats by rescuing the oxidative stress and inflammation and promoting BDNF releasing. Mol. Neurobiol. 53, 5664–5670. 10.1007/s12035-015-9438-1 26482461

[B20] Joshi C. R. Stacy S. Sumien N. Ghorpade A. Borgmann K. (2020). Astrocyte HIV-1 tat differentially modulates behavior and brain MMP/TIMP balance during short and prolonged induction in transgenic mice. Front. Neurol. 11:593188. 10.3389/fneur.2020.593188 33384653 PMC7769877

[B21] Ju X. Mallet R. T. Downey H. F. Metzger D. B. Jung M. E. (2012). Intermittent hypoxia conditioning protects mitochondrial cytochrome c oxidase of rat cerebellum from ethanol withdrawal stress. J. Appl. Physiol. 112, 1706–1714. 10.1152/japplphysiol.01428.2011 22403345 PMC3365408

[B22] Jung M. E. Simpkins J. W. Wilson A. M. Downey H. F. Mallet R. T. (2008). Intermittent hypoxia conditioning prevents behavioral deficit and brain oxidative stress in ethanol-withdrawn rats. J. Appl. Physiol. 105, 510–517. 10.1152/japplphysiol.90317.2008 18499779 PMC2519950

[B23] Kim J. Lee S. Choi B. R. Yang H. Hwang Y. Park J. H.. (2017). Sulforaphane epigenetically enhances neuronal BDNF expression and TrkB signaling pathways. Mol. Nutr. Food Res. 61:194. 10.1002/mnfr.201600194 27735126

[B24] Klein A. Kowall N. Ferrante R. (1998). Neurotoxicity and oxidative damage of beta amyloid 1-42 versus beta amyloid 1-40 in the mouse cerebral cortex. Ann. N. Y. Acad. Sci. 893, 314–320. 10.1111/j.1749-6632.1999.tb07845.x 10672257

[B25] Kuo C. Y. Hsiao H. T. Lo I. H. Nikolai T. (2020). Association between obstructive sleep apnea, its treatment, and alzheimer's disease: systematic mini-review. Front. Aging Neurosci. 12:591737. 10.3389/fnagi.2020.591737 33488381 PMC7815938

[B26] Lai S. W. Chen J. H. Lin H. Y. Liu Y. S. Tsai C. F. Chang P. C.. (2018). Regulatory effects of neuroinflammatory responses through brain-derived neurotrophic factor signaling in microglial cells. Mol. Neurobiol. 55, 7487–7499. 10.1007/s12035-018-0933-z 29427085

[B27] Li S. Hafeez A. Noorulla F. Geng X. Shao G. Ren C.. (2017). Preconditioning in neuroprotection: from hypoxia to ischemia. Prog. Neurobiol. 157, 79–91. 10.1016/j.pneurobio.2017.01.001 28110083 PMC5515698

[B28] Li Y. Lu Z. Keogh C. L. Yu S. P. Wei L. (2007). Erythropoietin-induced neurovascular protection, angiogenesis, and cerebral blood flow restoration after focal ischemia in mice. J. Cereb. Blood Flow Metab. 27, 1043–1054. 10.1038/sj.jcbfm.9600417 17077815

[B29] Liu X. Xu D. Hall J. R. Ross S. Chen S. Liu H.. (2017). Enhanced cerebral perfusion during brief exposures to cyclic intermittent hypoxemia. J. Appl. Physiol. 123, 1689–1697. 10.1152/japplphysiol.00647.2017 29074711

[B30] Lukyanova L. D. Sukoyan G. V. Kirova Y. I. (2013). Role of proinflammatory factors, nitric oxide, and some parameters of lipid metabolism in the development of immediate adaptation to hypoxia and HIF-1α accumulation. Bull. Exp. Biol. Med. 154, 597–601. 10.1007/s10517-013-2008-5 23658877

[B31] Malhotra S. Savitz S. I. Ocava L. Rosenbaum D. M. (2006). Ischemic preconditioning is mediated by erythropoietin through PI-3 kinase signaling in an animal model of transient ischemic attack. J. Neurosci. Res. 83, 19–27. 10.1002/jnr.20705 16307446

[B32] Mallet R. T. Ryou M. G. (2017). Erythropoietin: endogenous protection of ischemic brain. Vitam. Horm. 105, 197–232. 10.1016/bs.vh.2017.01.002 28629519

[B33] Mallet R. T. Ryou M. G. Williams A. G. Jr Howard L. Downey H. F. (2006). Beta1-Adrenergic receptor antagonism abrogates cardioprotective effects of intermittent hypoxia. Basic Res. Cardiol. 101, 436–446. 10.1007/s00395-006-0599-y 16705468

[B34] Miranda M. Morici J. F. Zanoni M. B. Bekinschtein P. (2019). Brain-derived neurotrophic factor: a key molecule for memory in the healthy and the pathological brain. Front. Cell Neurosci. 13:363. 10.3389/fncel.2019.00363 31440144 PMC6692714

[B35] Navarrete-Opazo A. Mitchell S. G. (2014). Therapeutic potential of intermittent hypoxia: a matter of dose. Am. J. Physiol. Regul. Integr. Comp. Physiol. 307, R1181–1197. 10.1152/ajpregu.00208.2014 25231353 PMC4315448

[B36] Ontiveros-Torres M. Labra-Barrios M. L. Díaz-Cintra S. Aguilar-Vázquez A. R. Moreno-Campuzano S. Flores-Rodríguez P.. (2016). Fibrillar amyloid-β accumulation triggers an inflammatory mechanism leading to hyperphosphorylation of the carboxyl-terminal end of tau polypeptide in the hippocampal formation of the 3 × Tg-AD transgenic mouse. J. Alzheimers Dis. 52, 243–269. 10.3233/JAD-150837 27031470

[B37] Park H. Poo M. M. (2013). Neurotrophin regulation of neural circuit development and function. Nat. Rev. Neurosci. 14, 7–23. 10.1038/nrn3379 23254191

[B38] Phillips S. A. Olson E. B. Morgan B. J. Lombard J. H. (2004). Chronic intermittent hypoxia impairs endothelium-dependent dilation in rat cerebral and skeletal muscle resistance arteries. Am. J. Physiol. Heart Circ. Physiol. 286, H388–393. 10.1152/ajpheart.00683.2003 14512283

[B39] Pillai A. Dhandapani K. M. Pillai B. A. Terry A. V. Mahadik P. S. Jr (2008). Erythropoietin prevents haloperidol treatment-induced neuronal apoptosis through regulation of BDNF. Neuropsychopharmacology 33, 1942–1951. 10.1038/sj.npp.1301566 17805306

[B40] Rabie T. Marti H. H. (2008). Brain protection by erythropoietin: a manifold task. Physiology 23, 263–274. 10.1152/physiol.00016.2008 18927202

[B41] Ratilal B. O. Arroja M. M. Rocha J. P. Fernandes A. M. Barateiro A. P. Brites D. M.. (2014). Neuroprotective effects of erythropoietin pretreatment in a rodent model of transient middle cerebral artery occlusion. J. Neurosurg. 121, 55–62. 10.3171/2014.2.JNS132197 24702327

[B42] Rey F. Balsari A. Giallongo T. Ottolenghi S. Di Giulio A. M. Samaja M.. (2019). Erythropoietin as a neuroprotective molecule: an overview of its therapeutic potential in neurodegenerative diseases. ASN Neuro 11:1759091419871420. 10.1177/1759091419871420 31450955 PMC6712762

[B43] Romberg C. Mattson M. P. Mughal M. R. Bussey T. J. Saksida L. M. (2011). Impaired attention in the 3xTgAD mouse model of Alzheimer's disease: rescue by donepezil (Aricept). J. Neurosci. 31, 3500–3507. 10.1523/JNEUROSCI.5242-10.2011 21368062 PMC3066152

[B44] Ruscher K. Freyer D. Karsch M. Isaev N. Megow D. Sawitzki B.. (2002). Erythropoietin is a paracrine mediator of ischemic tolerance in the brain: evidence from an *in vitro* model. J. Neurosci. 22, 10291–10301. 10.1523/JNEUROSCI.22-23-10291.2002 12451129 PMC6758760

[B45] Rybnikova E. Samoilov M. (2015). Current insights into the molecular mechanisms of hypoxic pre- and postconditioning using hypobaric hypoxia. Front. Neurosci. 9:388. 10.3389/fnins.2015.00388 26557049 PMC4615940

[B46] Ryou M. G. Liu R. Ren M. Sun J. Mallet R. T. Yang S. H. (2012). Pyruvate protects the brain against ischemia-reperfusion injury by activating the erythropoietin signaling pathway. Stroke 43, 1101–1107. 10.1161/STROKEAHA.111.620088 22282883 PMC3314723

[B47] Ryou M. G. Mallet R. T. Metzger D. B. Jung M. E. (2017). Intermittent hypoxia training blunts cerebrocortical presenilin 1 overexpression and amyloid-beta accumulation in ethanol-withdrawn rats. Am. J. Physiol. Regul. Integr. Comp. Physiol. 313, R10–R18. 10.1152/ajpregu.00050.2017 28490448 PMC5538853

[B48] Sakanaka M. Wen T. C. Matsuda S. Masuda S. Morishita E. Nagao M.. (1998). *In vivo* evidence that erythropoietin protects neurons from ischemic damage. Proc. Natl. Acad. Sci. U. S. A. 95, 4635–4640. 10.1073/pnas.95.8.4635 9539790 PMC22542

[B49] Semenza G. L. (2009). Regulation of oxygen homeostasis by hypoxia-inducible factor 1. Physiology 24, 97–106. 10.1152/physiol.00045.2008 19364912

[B50] Shingo T. Sorokan S. T. Shimazaki T. Weiss S. (2001). Erythropoietin regulates the *in vitro* and *in vivo* production of neuronal progenitors by mammalian forebrain neural stem cells. J. Neurosci. 21, 9733–9743. 10.1523/JNEUROSCI.21-24-09733.2001 11739582 PMC6763035

[B51] Shiota S. Takekawa H. Matsumoto S. E. Takeda K. Nurwidya F. Yoshioka Y.. (2013). Chronic intermittent hypoxia/reoxygenation facilitate amyloid-beta generation in mice. J. Alzheimers Dis. 37, 325–333. 10.3233/JAD-130419 23948880

[B52] Sisodia S. S. Price L. D. (1995). Role of the beta-amyloid protein in Alzheimer's disease. Faseb J. 9, 366–370. 10.1096/fasebj.9.5.7896005 7896005

[B53] Song J. H. Yu J. T. Tan L. (2015). Brain-derived neurotrophic factor in Alzheimer's disease: risk, mechanisms, and therapy. Mol. Neurobiol. 52, 1477–1493. 10.1007/s12035-014-8958-4 25354497

[B54] Tahawi Z. Orolinova N. Joshua I. G. Bader M. Fletcher E. C. (2001). Altered vascular reactivity in arterioles of chronic intermittent hypoxic rats. J. Appl. Physiol. 90, 2007–2013; discussion 2000. 10.1152/jappl.2001.90.5.2007 11299297

[B55] Vetrovoy O. V. Rybnikova E. A. Samoilov M. O. (2017). Cerebral mechanisms of hypoxic/ischemic postconditioning. Biochemistry 82, 392–400. 10.1134/S000629791703018X 28320281

[B56] Villa P. Bigini P. Mennini T. Agnello D. Laragione T. Cagnotto A.. (2003). Erythropoietin selectively attenuates cytokine production and inflammation in cerebral ischemia by targeting neuronal apoptosis. J. Exp. Med. 198, 971–975. 10.1084/jem.20021067 12975460 PMC2194205

[B57] Viviani B. Bartesaghi S. Corsini E. Villa P. Ghezzi P. Garau A.. (2005). Erythropoietin protects primary hippocampal neurons increasing the expression of brain-derived neurotrophic factor. J. Neurochem. 93, 412–421. 10.1111/j.1471-4159.2005.03033.x 15816864

[B58] Wall A. M. Corcoran A. E. O'Halloran K. D. O'Connor J. J. (2014). Effects of prolyl-hydroxylase inhibition and chronic intermittent hypoxia on synaptic transmission and plasticity in the rat CA1 and dentate gyrus. Neurobiol. Dis. 62, 8–17. 10.1016/j.nbd.2013.08.016 24055213

[B59] Wang H. Shi X. Schenck H. Hall J. R. Ross S. E. Kline G. P.. (2020). Intermittent hypoxia training for treating mild cognitive impairment: a pilot study. Am. J. Alzheimers Dis. Other Demen. 35:1533317519896725. 10.1177/1533317519896725 31902230 PMC10624018

[B60] Wang L. Zhang Z. Wang Y. Zhang R. Chopp M. (2004). Treatment of stroke with erythropoietin enhances neurogenesis and angiogenesis and improves neurological function in rats. Stroke 35, 1732–1737. 10.1161/01.STR.0000132196.49028.a4 15178821

[B61] Wu C. L. Chen S. D. Yin J. H. Hwang C. S. Yang I. D. (2010). Erythropoietin and sonic hedgehog mediate the neuroprotective effects of brain-derived neurotrophic factor against mitochondrial inhibition. Neurobiol. Dis. 40, 146–154. 10.1016/j.nbd.2010.05.019 20580927

[B62] Yamada K. Nabeshima T. (2003). Brain-derived neurotrophic factor/TrkB signaling in memory processes. J. Pharmacol. Sci. 91, 267–270. 10.1254/jphs.91.267 12719654

[B63] Yang J. L. Lin Y. T. Chuang P. C. Bohr V. A. Mattson M. P. (2014). BDNF and exercise enhance neuronal DNA repair by stimulating CREB-mediated production of apurinic/apyrimidinic endonuclease 1. Neuromolecular Med. 16, 161–174. 10.1007/s12017-013-8270-x 24114393 PMC3948322

[B64] Zhang P. Downey H. F. Chen S. Shi X. (2014). Two-week normobaric intermittent hypoxia exposures enhance oxyhemoglobin equilibrium and cardiac responses during hypoxemia. Am. J. Physiol. Regul. Integr. Comp. Physiol. 307, R721–730. 10.1152/ajpregu.00191.2014 25056104

[B65] Zhu X. H. Yan H. C. Zhang J. Qu H. D. Qiu X. S. Chen L.. (2010). Intermittent hypoxia promotes hippocampal neurogenesis and produces antidepressant-like effects in adult rats. J. Neurosci. 30, 12653–12663. 10.1523/JNEUROSCI.6414-09.2010 20861371 PMC6633584

[B66] Zong P. Setty S. Sun W. Martinez R. Tune J. D. Ehrenburg I. V.. (2004). Intermittent hypoxic training protects canine myocardium from infarction. Exp. Biol. Med. 229, 806–812. 10.1177/153537020422900813 15337835

[B67] Zuccato C. Marullo M. Conforti P. MacDonald M. E. Tartari M. Cattaneo E. (2008). Systematic assessment of BDNF and its receptor levels in human cortices affected by Huntington's disease. Brain Pathol. 18, 225–238. 10.1111/j.1750-3639.2007.00111.x 18093249 PMC8095509

